# A Prediction Model for Lymph Node Metastasis of Oral Squamous Cell Carcinoma Based on Multiple Risk Factors

**DOI:** 10.1002/cre2.70046

**Published:** 2024-11-17

**Authors:** Hongyu Shen, Tonghan Zhang, Shuoyu Wang

**Affiliations:** ^1^ School of Stomatology Jinan University Guangzhou China; ^2^ Department of Oral and Maxillofacial Surgery Hospital of Stomatology Zhongshan China

**Keywords:** cervical lymph node metastasis, oral squamous cell carcinoma, prediction model, risk factors

## Abstract

**Objectives:**

Cervical lymph node metastasis (CLNM) frequently occurs in oral cancer patients. This study aims to investigate risk factors associated with CLNM and predict CLNM preoperatively in patients with oral squamous cell carcinoma (OSCC).

**Materials and Methods:**

This population‐based, hospital retrospective cohort study included 158 patients with oral cancer. We performed regression analysis to determine risk factors and establish a model for predicting CLNM in patients with OSCC. To distinguish and validate the prediction model, we used the area under the receiver operating characteristic (ROC) curve (AUC).

**Results:**

Lymph node size, tumor size, degree of differentiation, and LVI were risk factors for cancer metastasis. The OR values were 1.245, 2.847, 2.527, and 6.945, respectively. The AUC value for the clinical prediction model was 0.8736 (95% CI: 0.8043–0.9429).

**Conclusions:**

The prediction model for OSCC patients predicts CLNM and provides a new method for preoperative assessment of whether cervical lymph nodes are metastatic, as well as a guide for surgical treatment, including whether to carry out neck dissection and which neck dissection procedure to use.

## 1 Introduction

Malignant tumors in the oral and maxillofacial areas are most commonly represented by cancer, including tongue cancer, gingival cancer, cheek cancer, palate cancer, floor‐of‐mouth cancer, carcinoma of the lip, and carcinoma of the oropharynx (Zhang et al. 2020). Among them, squamous cell carcinoma is the most prevalent pathological kind, with a 5‐year survival rate of approximately 55% (Liu et al. 2024). The prognosis of patients is significantly influenced by the metastasis of cervical lymph nodes (Reticker‐Flynn and Engleman 2023). Cervical lymph node metastasis (CLNM) can result in cancers that spread and may have a direct effect on patients’ quality of life or be directly linked to their mortality. Therefore, neck dissection has become an important component of treatment for patients with oral squamous cell carcinoma (OSCC; Abdullah Zubir et al. 2020). According to the oral and oropharyngeal cancer section of the NCCN Guidelines (Pfister et al. 2024), it is recommended to consider primary tumor resection with or without (±) ipsilateral neck dissection for T1–T2, N0 oral cancer patients, or bilateral neck dissection. Another option is primary tumor resection plus sentinel lymph node biopsy, with additional therapy decided upon by the biopsy findings. However, some OSCC patients suffer occult CLNM. For example, tongue cancer has a metastasis rate of 20%–40% (Sano and Myers 2007). Therefore, in clinical practice, we often encounter some patients who initially show negative CLNM after clinical examination and imaging examination (cN0) but are later diagnosed as positive for CLNM through pathological examination after surgery (pN+) (Thiele et al. 2012). Given the aforementioned circumstances, accurately determining the status of CLNM before surgery has become a pressing issue to be solved (Hung et al. 2022).

Our purpose was to investigate risk factors associated with CLNM in patients with oral cancer. We hypothesize that age, gender, lymph node size, tumor size, T stage, differentiation grade, lymphovascular invasion (LVI), and perineural invasion (PNI) are associated with CLNM and will subject them to statistical analysis. The utilization of nomograms in cancer prognosis is widely adopted due to their ability to simplify predictive models into numerical estimates of probabilities for specific events such as death or metastasis (Iasonos et al. 2008). Therefore, the risk factors with statistical significance were drawn into the nomogram to achieve the ultimate aims of preoperative prediction of CLNM.

## 2 Materials and Methods

### 2.1 Study Design

A retrospective collection of 158 patients was diagnosed with oral cancer by pathological examination and had resection of primary plus neck dissection at the Department of Oral and Maxillofacial Surgery, Zhongshan People's Hospital, from October 2020 to October 2023. Zhongshan People's Hospital gave its approval for this study to be conducted retrospectively; thus, informed consent was not required. The R4.3.2 random sampling method was used to divide 158 cases into the training group and validation group according to a 4:1 ratio.

The inclusion criteria were as follows: (1) patients diagnosed with oral cancer through preoperative pathological examination; (2) patients who had not received any other treatments such as radiotherapy or chemotherapy; (3) patients who underwent various biochemical tests, such as electrocardiography, chest X‐rays, CT scans, and MRI scans, before surgery; (4) patients who underwent neck dissection and had routine pathological biopsy conducted during surgery; (5) patients with complete clinical, pathological, and imaging data. The exclusion criteria were as follows: (1) combined with tumors in other parts of the body; (2) previous surgical history of head and neck tumors and recurrence; and (3) poor general condition, unable to tolerate surgical treatment.

### 2.2 Data Collection

Clinical data of patients were collected: patient's name, hospitalization number, age, gender, lymph node size, T stage, tumor size, differentiation degree, LVI, and PNI. The imaging examination criteria for CLNM are met if any of the subsequent circumstances apply: lymph node size in Zones I–II is ≥ 11 mm, and in Zones III–IV is ≥ 10 mm; liquefaction necrosis appears centrally, and calcification appears within the lymph nodes; there are three or more lymph node clusters around the primary tumor and its associated lymph drainage area (Jones et al. 1994). In this study, lymph node size was measured using MRI imaging (Philips Achieva3.0 T, 2008) from the Netherlands.

### 2.3 Statistical Analysis

The data analysis software utilized was SPSS 27.0. The lymph node size, tumor size, and patient's age were all tested using independent sample *t*‐tests. The differences in T stage, differentiation degree, LVI, and PNI between the two groups were compared using the chi‐square test. The regression analysis consisted of univariate and multivariate regression analyses. Age, gender, tumor size, lymph node size, T stage, differentiation degree, LVI, and PNI were included in univariate regression analysis to screen out statistically significant factors. The above‐selected risk factors were incorporated into a multivariate regression analysis to screen the final risk factors. When *p* < 0.05, it is considered that the screened risk factors have statistical significance. The R software (version 4.3.2) was used to draw a nomogram through the screened risk factors, and the models were distinguished and validated using the ROC curve and the AUC.

## 3 Results

### 3.1 Clinical Information

Table 1 displays the clinical information of 158 patients, with an average age of 55.05 ± 13.31 years. Following testing, no statistically significant variations in any of the variables were found between the two groups, such as tumor size, lymph node size, T stage, differentiation degree, LVI, PNI, and age (*p* > 0.05), all of which could be included in logistics regression analysis.

**Table 1:** Patients' clinical information.

Group	Training group (*n* = 126)	Validation group (*n* = 32)	*t*/*χ* ^2^	*p*
Age, *n* (year)	55.37 ± 13.68	53.81 ± 11.67	0.586	0.458
Gender, *n* (%)			0.653	0.419
Male	81 (64.3)	23 (71.9)		
Female	45 (35.7)	9 (28.1)		
Differentiation grade, *n* (%)			2.077	0.354
Well	68 (54.0)	13 (40.6)		
Moderately	53 (42.1)	18 (56.3)		
Poorly	5 (3.9)	1 (3.1)		
T stage, *n* (%)			0.361	0.948
T1	59 (46.8)	15 (46.9)		
T2	44 (35.0)	11 (34.4)		
T3	18 (14.3)	4 (12.5)		
T4	5 (3.9)	2 (6.2)		
Tumor size, *n* (mm)	2.13 ± 1.25	2.09 ± 1.25	0.195	0.584
Lymph node size, *n* (mm)	6.03 ± 4.90	7.53 ± 4.86	−1.53	0.936
LVI, *n* (%)			0.02	0.887
No	105 (83.3)	27 (84.4)		
Yes	21 (16.7)	5 (15.6)		
PNI, *n* (%)			0.049	0.825
No	93 (73.8)	23 (71.9)		
Yes	33 (26.2)	9 (28.1)		
Metastasis, *n* (%)				
No	85 (67.5)	17 (53.1)		
Yes	41 (32.5)	15 (46.9)		

### 3.2 Logistic Regression Analysis Results

Age, gender, tumor size, lymph node size, T stage, differentiation degree, LVI, and PNI were taken as independent variables, and whether lymph node metastasis occurred served as the dependent variable. The results were shown after univariate logistic regression analysis (as shown in Table 2). Tumor size, lymph node size, T stage, differentiation degree, LVI, and PNI were identified as statistical risk factors for cancer metastasis in oral cancer (*p* < 0.05).

**Table 2:** Results of univariate logistic regression analysis of lymph node metastasis.

Risk factors	OR	95% CI	*p*
Age	1.008	0.981–1.036	0.578
Gender	0.652	0.292–1.454	0.296
Differentiation degree	2.268	1.171–4.394	0.015
T stage	2.161	1.356–3.445	0.001
Tumor size	2.243	1.530–3.290	< 0.001
Lymph node size	1.259	1.139–1.392	< 0.001
LVI	10.240	3.409–30.763	< 0.001
PNI	2.560	1.125–5.826	0.025

The above‐screened risk factors were taken as independent variables for multivariate logistic regression analysis. The results showed (as shown in Table 3) that differentiation degree, tumor size, lymph node size, and LVI were independent risk factors that ultimately affected cancer metastasis in oral cancer. The OR values were 2.527 (95% CI: 1.067–5.987), 2.847 (95% CI: 1.424–5.693), 1.245 (95% CI: 1.108–1.398), and 6.945 (95% CI: 1.836–26.262), respectively.

**Table 3:** Results of multivariate logistic regression analysis of lymph node metastasis.

Risk factors	OR	95% CI	*p*
Differentiation degree	2.527	1.067–5.987	0.035
Tumor size	2.847	1.424–5.693	0.003
Lymph node size	1.245	1.108–1.398	< 0.001
LVI	6.945	1.836–26.262	0.004

### 3.3 Establishment and Validation of Nomogram

**Figure 1:**
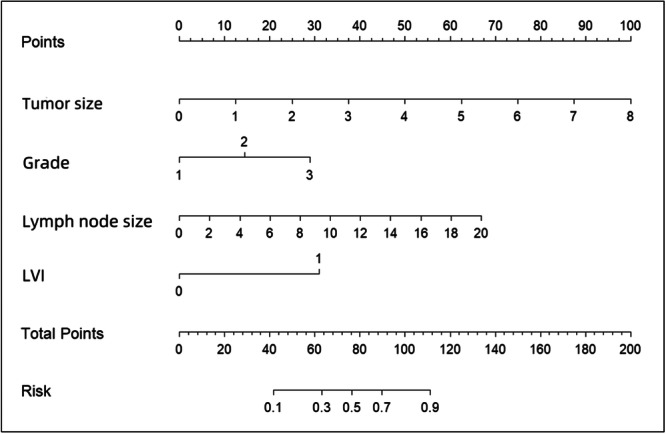
The nomogram prediction model for lymph node metastasis in oral cancer patients.

**Figure 2:**
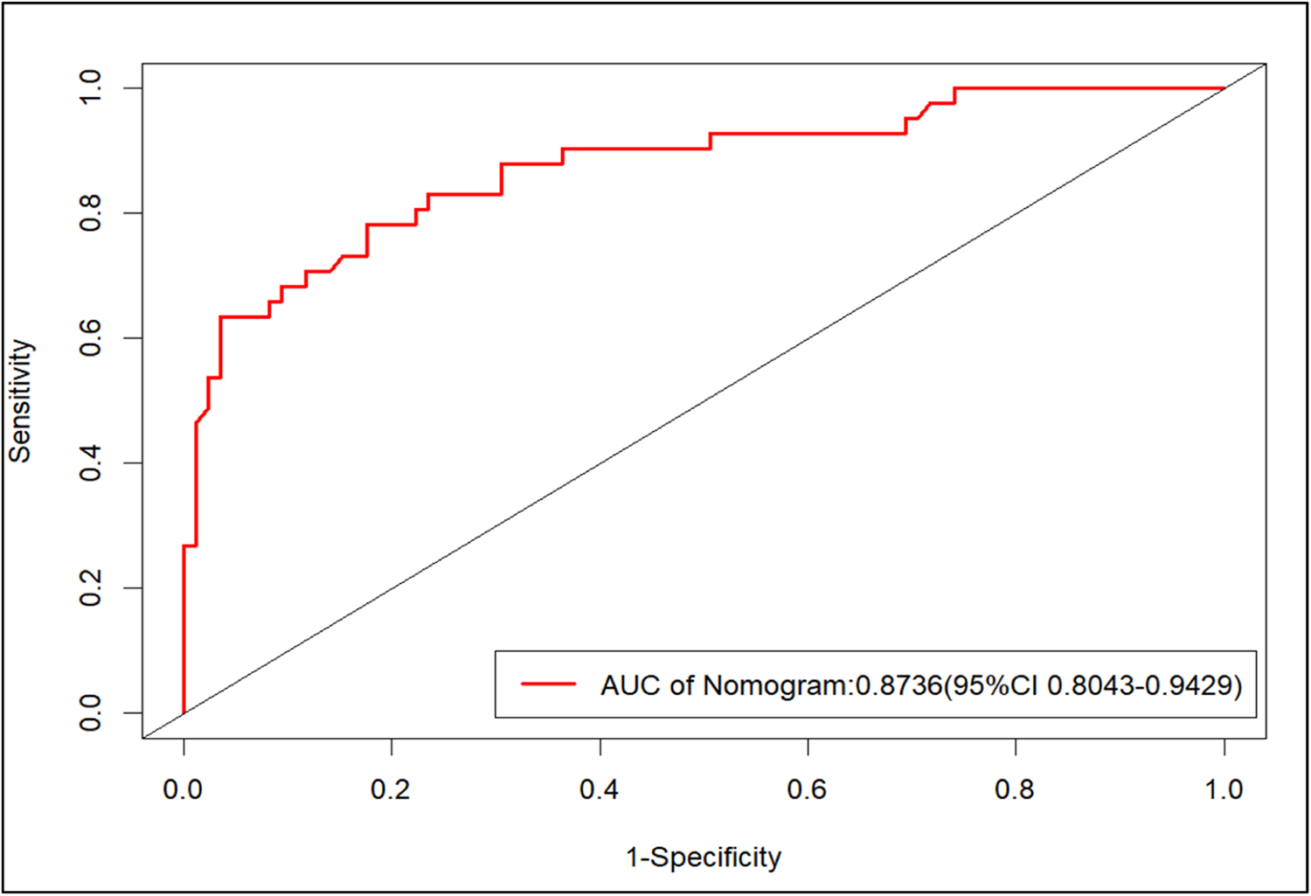
The ROC curve of the clinical prediction model.

**Figure 3:**
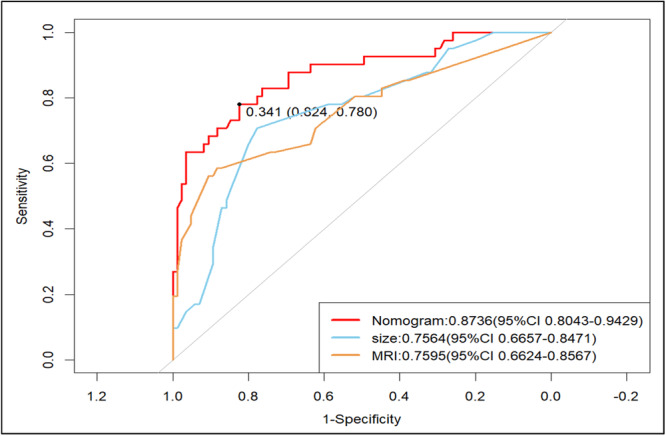
The ROC curve of the clinical prediction model, with tumor size and lymph node size. The red line represents the clinical prediction model, the blue line represents the tumor size model, and the yellow line represents the lymph node size model.

**Figure 4:**
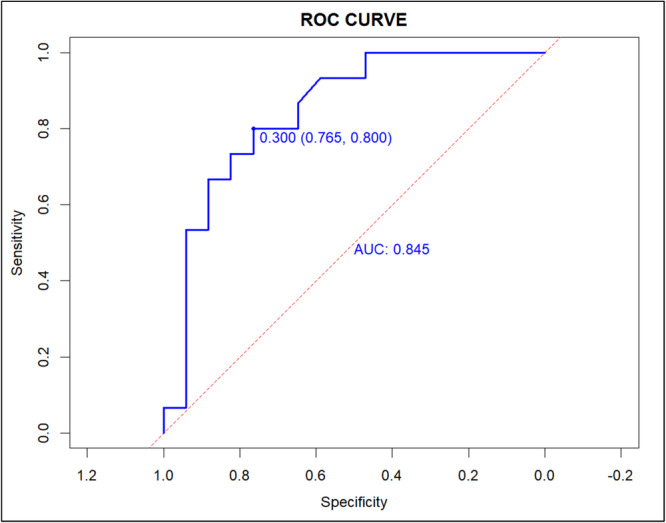
The ROC curve of the validation group.

Based on the independent risk factors (tumor size, lymph node size, differentiation degree, and LVI) assessed by multivariate logistic regression analysis, a nomogram was established (Figure 1). The ROC curve of the clinical prediction model, which has an AUC value of 0.8736 (95% CI: 0.8043–0.9429), is displayed in Figure 2. Figure 3 displays a comparison of the ROC curves for the three models. The AUC values for the lymph node size and tumor size prediction models were 0.7595 (95% CI: 0.6624–0.8567) and 0.7564 (95% CI: 0.6657–0.8471), respectively. The specificity and sensitivity of the model (Table 4) showed that the specificity, sensitivity, and AUC of the clinical model were higher than those of the other two models. Figure 4 displays the ROC curve of the validation model, and its specificity, sensitivity, and AUC values are 0.765, 0.800, and 0.845, respectively. The decision curve and calibration curve are shown in Graph 1 and Graph 2 (Supporting Information).

**Table 4:** Predictive performance of three models.

	Sensitivity	Specificity	AUC	PPV	NPV
Clinical model	0.780	0.824	0.8736	0.6310	0.8859
Tumor size model	0.707	0.776	0.7564	0.5379	0.8459
Lymph node size model	0.585	0.882	0.7595	0.4047	0.8128

## 4 Discussion

The purpose of this study was to explore risk factors associated with CLNM and establish a predictive model. The sixth most frequent cancer worldwide is head and neck cancer. More than 50% of these cases involve oral cancer. Of these, OSCC is the most frequent, accounting for 90% of occurrences (Johnson et al. 2020; Warnakulasuriya and Kerr 2021; Radaic et al. 2023). Oral cancer often metastasizes early, primarily to the cervical lymph nodes. However, it can also reach the brain, bones, and lungs through the bloodstream, among other bodily regions. Once metastasis occurs, it significantly affects the course of treatment and the prognosis for the patient's illness. In patients with oral cancer, we generally perform surgery based on the patient's tumor size and the lymph node size observed on imaging. However, some patients may experience occult CLNM. For instance, 20%–30% of early‐stage tongue carcinoma (T1/2, N0) cases have occult metastases (Monroe and Gross 2012). However, for those with tongue cancer at the T3/4 stage, we perform extended resection of the primary plus neck dissection, which is a noncontroversial issue, and finally choose whether to perform further flap repair based on the size of the defect site. Therefore, it is still an unsolved problem to accurately determine whether CLNM occurs before surgery. Both blind neck dissection and conservative treatment may potentially harm patients. For instance, complications that may arise after neck dissection include harm to the accessory nerve, and the marginal mandibular branch of the facial nerve, in addition to esthetic issues caused by the incision (de Bree et al. 2019). In this study, we identified tumor size, degree of differentiation, lymph node size, and LVI as independent risk factors and established a model for preoperative prediction of CLNM to provide clinical treatment for oral cancer patients.

For OSCC patients, the following risk factors influence the metastasis of cervical lymph nodes: tumor size, T stage, differentiation degree, PNI, extranodal expansion (ENE), and LVI (Liu et al. 2024; Pfister et al. 2024; Li et al. 2019; Essig et al. 2012). Literature (Wang et al. 2022) has shown that lymph node size can be used as an independent variable to predict cancer metastasis. In addition, the general consensus in clinical practice is that the likelihood of metastasis increases with lymph node size. Therefore, we consider it an independent risk factor. The larger the tumor, the greater the possible depth and scope of invasion, and the greater the possibility of occult metastasis (Al‐Rajhi et al. 2000). However, studies by d'Alessandro et al. (2015) and Morton et al. (1994) contradicted our study's results. We think that the inability to identify the tumor's area of occurrence may be the cause of the inconsistent outcomes. The prognosis worsens with a decreasing pathogenic differentiation degree and the absence of cancer adhesion factors in tumor cells (Wu et al. 2016). Our study's results are in line with those of Wu et al. (2016) and Kademani et al. (2005), who also found similar results. However, some studies have found that the pathological differentiation degree and CLNM are not substantially associated (Woolgar 2006).

A crucial stage in the LVI‐metastasis cascade is the presence of malignant cells in the LVI system. When LVI is detected in the area surrounding the tumor, it is considered to be an indicator affecting the incidence and metastasis of cancer and is strongly linked to the poor prognosis of cancer (Aleskandarany et al. 2015). Mascitti et al. (2022) reported that LVI was not related to tumor prognosis but was linked with lymph node metastasis and tumor recurrence. Huang et al. (2021) suggested that LVI could be used as an independent indicator of lymph node metastasis and prognosis in patients.

Additionally, in this study, the univariate logistic regression analysis revealed that the T stage was statistically significant. However, after being included in the multivariate logistic regression analysis, it was found to be nonsignificant. This may be caused by the interaction between variables. Therefore, we decided to exclude it.

A nomogram can visually represent the logistic regression results we desire more intuitively. In OSCC patients, we found a relationship between CLNM and tumor size, lymph node size, differentiation degree, and LVI. These results have been compiled and presented in a nomogram for visual representation. A score for each of these risk factors is obtained by the physician, and the score is then added together to obtain the final score, which corresponds to the risk of metastasis in patients. Compared to traditional judgment, the nomogram can predict the status of lymph nodes precisely; so this tool can be tried to be applied to clinical treatment. The limitations of this study are the small sample size and the fact that it is a retrospective study, which may be influenced by inherent data. Multi‐center research with large sample sizes is of greater value to this study.

This study established a nomogram model of risk variables for lymph node metastasis, which can predict the status of lymph nodes in OSCC patients before surgery, assist doctors in clinical decision‐making, and provide a new method for accurate diagnosis and treatment.

## Author Contributions

Hongyu Shen contributed to the conceptualization and design of the study, performed data collection and analysis, and drafted the manuscript. Tonghan Zhang contributed to the conception and design of the study and revised the manuscript. Shuoyu Wang contributed to the investigation of the study and validated the data results.

## Conflicts of Interest

The authors declare no conflicts of interest.

## Supporting information

Supporting information.

Supporting information.

Supporting information.

Supporting information.

Supporting information.

## Data Availability

The data that support the findings of this study are available in the Supporting Information of this article. The data that support the findings of this study are available on request from the corresponding author. The data are not publicly available due to privacy or ethical restrictions.
