# Supplementary material for: A Prediction Model for Lymph Node Metastasis of Oral Squamous Cell Carcinoma Based on Multiple Risk Factors
Source: Clin Exp Dent Res. 2024 Nov 17;10(6):e70046. doi: 10.1002/cre2.70046 (PMC11570548; doi:10.1002/cre2.70046)
Supplement: Supplementary file 1 — Supporting information. [file CRE2-10-e70046-s004.docx]

Graph1: The calibration curve demonstrates that there is an average error of 0.027 between the model's actual and predicted values. The predicted risk is similar to the actual risk, suggesting that the predictive model has good performance.

Graph2: The decision curve shows that as the risk threshold increases, the net return of the model decreases.
