# Supplementary figures and images for: A Prediction Model for Lymph Node Metastasis of Oral Squamous Cell Carcinoma Based on Multiple Risk Factors
Source: Clin Exp Dent Res. 2024 Nov 17;10(6):e70046. doi: 10.1002/cre2.70046 (PMC11570548; doi:10.1002/cre2.70046)

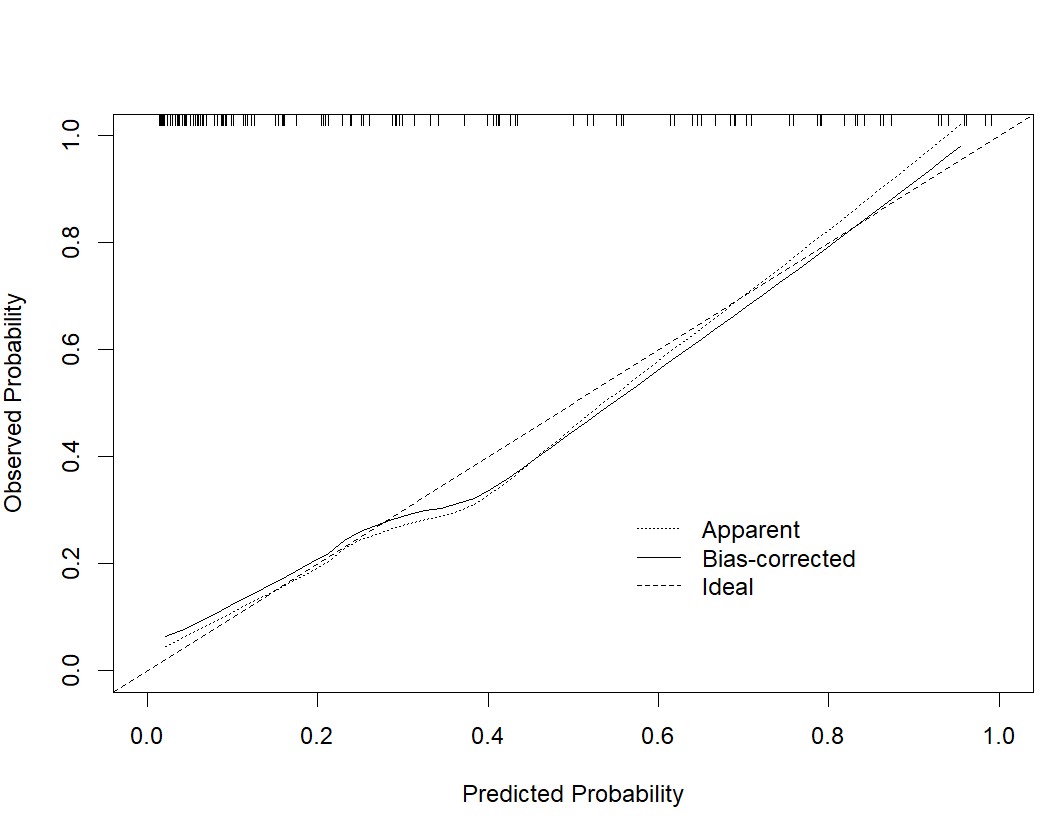

Supplement: Supplementary file 2 — Supporting information. [file CRE2-10-e70046-s003.jpg]

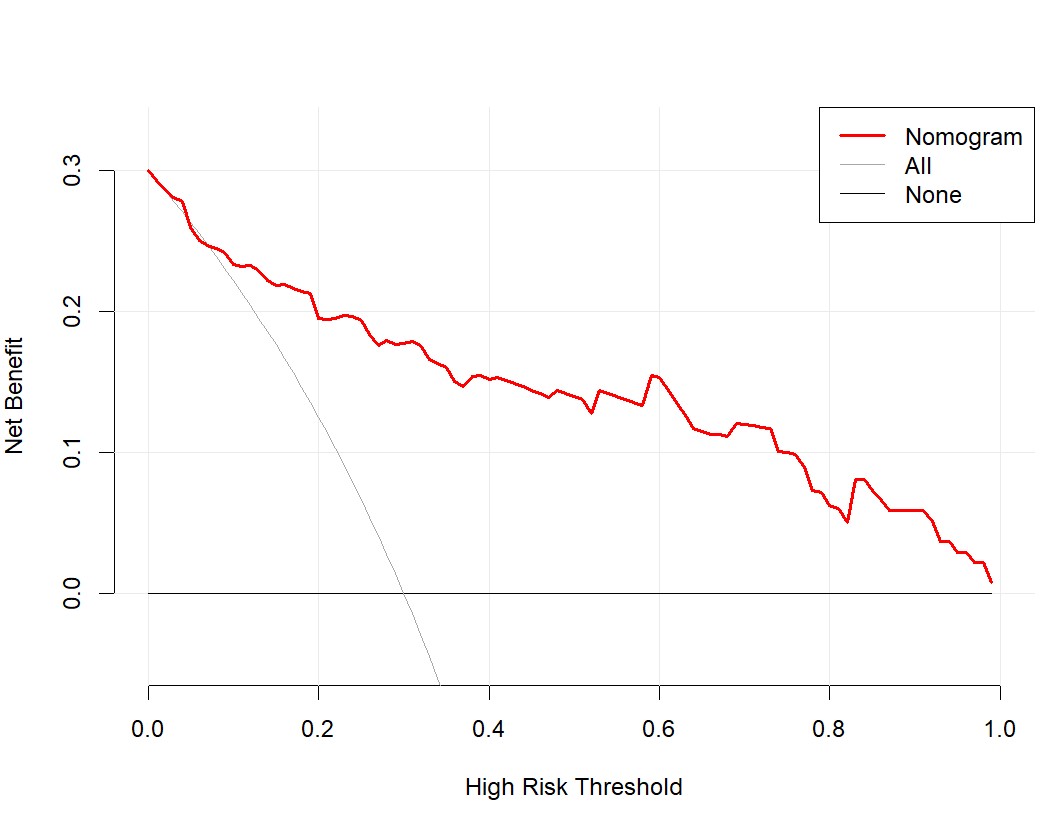

Supplement: Supplementary file 3 — Supporting information. [file CRE2-10-e70046-s002.jpg]
